# Modulation of RANTES expression by HCV core protein in liver derived cell lines

**DOI:** 10.1186/1471-230X-7-21

**Published:** 2007-06-12

**Authors:** Anna Ruggieri, Marina Franco, Ilaria Gatto, Ajit Kumar, Maria Rapicetta

**Affiliations:** 1 Viral Hepatitis Unit, Department of Infectious, Parasitic and Immune-Mediated Diseases, Istituto Superiore di Sanità, Viale Regina Elena 299, 00161 Rome, Italy; 2 Department of Biochemistry and Molecular Biology and of Genetics, The George Washington University, 2300 I Street N.W., Washington D.C. 20037, USA

## Abstract

**Background:**

Hepatitis C virus (HCV) infection is associated with high percentage of chronicity which implies the ability of the virus to evade or modulate host cell immune system. Modulation of chemokines, such as RANTES may be part of the virus induced pathogenicity. We examined the effect of core and structural proteins of HCV on RANTES expression in two liver derived cell lines, HepG2 and Chang Liver (CHL).

**Methods:**

HepG2 and Chang Liver (CHL) cell lines were established and selected for constitutive expression of HCV core and structural genes. Flow cytometry and quantitative RT-PCR analysis were performed to examine the effect of HCV core protein on RANTES expression. Luciferase analysis after RANTES-Luc-promoter transfection of established cell lines was assayed by luminometer measurements (RLU) of RANTES promoter activity. IRF-1 and IRF-7 expression was then examined by immunoblotting analysis.

**Results:**

Results of flow cytometry and RT-PCR analysis indicated that RANTES is differentially regulated by HCV core protein in the two cell lines examined as its expression was inhibited in HepG2 cells, by a reduction of RANTES promoter activity. Conversely, RANTES protein and mRNA were induced by the core protein in CHL cells, through the induction of the promoter.

Since HCV genome modulates IRF-1 and IRF-7 in replicon system and IRF-1, IRF-3 and IRF-7 have been reported to regulate RANTES promoter in various cell systems, analysis of the mechanism underlying RANTES modulation by the core protein revealed that IRF-1 expression was induced in HepG2 cells by the core protein, whereas in CHL cells it was expressed at a very low level that was not influenced by transfection with the core protein construct. This suggested that IRF-1 level may mediate the expression of RANTES in cell lines of liver origin. The effect of the core protein on RANTES promoter was countered by co-transfection with NF90, a double-stranded-RNA binding protein that activates some interferon response genes and acts as a component of cell defense against viral infection.

**Conclusion:**

HCV core protein have opposite effects on the expression of RANTES in different cell types *in vitro*, possibly reflecting a similar scenario in different microenvironments *in vivo*.

## Background

Hepatitis C virus (HCV) is the major cause of chronic liver disease worldwide associated with the development of hepatocellular carcinoma (HCC), that is the fifth most common cancer worldwide [1], with increasing incidence in Europe [2] and the leading cause of death amongst cirrhotic patients [3,4].

One of the main features of HCV infection is the high percentage of chronicity which implies the ability of the virus to evade or modulate host cell immune system. Initial inflammatory response to early viral infection is associated to secretion of RANTES, a CC chemokine with chemotactic function which recruits circulating T cells to the inflammation sites. RANTES has been shown to be induced by several viral infections in a number of cell types, including the endothelial cell infection by HCMV [5]; in liver cells infected by dengue-2 virus [6], and in influenza A virus infection in monocyte/macrophages [7]. In tumors, a dual role of RANTES has been reported [8] since it may promote tumor progression, as in melanomas [9], or the spreading of tumor cells throughout the body, as in breast cancer [10]; but RANTES may also enhance immune response against tumors and associate to improved prognosis as in the case of lung cancer [11].

A possible role for RANTES/CCR5 pathways in the pathogenesis of chronic HCV infection has been suggested from studies on HCV patients. Polymorphism of RANTES promoter was reported to correlate with outcome of HCV associated disease [12,13], and individuals homozygous for deletion mutation of RANTES receptor (CCR5) showed increased susceptibility to chronic HCV infection [14]. Lichterfeld and co-workers [15] reported that T cells from HCV patients had reduced responsiveness to RANTES. In addition, intra-hepatic expression of RANTES and Mig is positively related to the severity of hepatic inflammation [16], and intra-hepatic levels of RANTES mRNA have been reported to increase in chronic HCV patients [17]. Modulation of RANTES expression in response to HCV has been somewhat controversial. *In vitro *studies have shown that full length HCV construct induces RANTES and MCP-1 expression in Huh7 and HeLa cells. On the other hand, inhibition of RANTES promoter in Huh7 and HepG2 cell lines has also been reported to be mediated by HCV core protein [18,19]. More recently interaction of HCV E2 protein and CD81 has been suggested to lead to increased RANTES secretion by CD8 + lymphocytes [15,20].

HCV core protein has been described as a multifunctional protein that in addition to function in viral nucleocapsid formation, influences several cellular function, such as cell growth and death [21-23], immune cells functions [24,25] and its expression in transgenic mice has been associated to development of steatosis and hepatocellular carcinoma [26,27]. Induction of chemokines by viral infection determines the local inflammation response and may be part of the virus induced pathogenicity. Modulation of chemokines expression by viruses or viral proteins may contribute to evasion of the innate immune response in early viral infection, affecting the outcome of infection and viral persistence.

RANTES activation by viral infection has been reported to be regulated by synergistic activity between interferon response factors, IRF-3, IRF-7 and NF-kB in various cell lines [28] and by IRF-1, which can bind to RANTES promoter in mouse macrophages [29]. Moreover, HCV genome has been reported to modulate IRF-1 and IRF-7 in replicon system [30,31].

NF90 belongs to a family of double-stranded-RNA binding proteins [32], which has been reported to regulate replication of the Pestivirus BVDV [33] (phylogenetically related to the Flavivirus family to which HCV belongs) and when introduced into osteosarcoma cell line it induces resistance to HIV replication in cell culture [34] acting as a component of cell defense against viral infection by activation of some IFN-response genes [34].

We examined the effect of HCV core and structural proteins on the expression of RANTES in human hepatoma and in a liver derived cell lines (HepG2 and Chang Liver) expressing different constructs of HCV genome. The expression of IRF-1 and IRF-7 has been examined to reveal their possible role in RANTES modulation by the core protein. In addition, we investigated the role of NF90 transfection on the expression of RANTES in order to reveal its possible immunomodulatory function and its potential to counteract HCV core protein effect on RANTES.

## Methods

### Stable transfectants and expression constructs

Stable transfectants in HepG2 cell lines expressing core and HCV structural proteins have been described by Harada et al., 1995 [35]. Stable transfectants of Chang Liver (CHL) cell lines expressing HCV core (CH39) or core-E1-E2-NS2-NS3 (CH352) were established by stable transfection of Chang Liver cells with pCAG39neo, pCAG352neo expression vectors respectively, that expressed HCV structural and non-structural proteins under the control of mammalian CAG promoter. Clonal selection of liver cell transfectants was performed in the presence of 1 mg/ml of G418 antibiotic. Expression levels of HCV proteins were monitored by Western blotting. Positive clones were cultured and maintained under G418 selection; however experiments were performed in G418-free medium to avoid the possible influence of the antibiotic on functions under study as well as to maintain homologous conditions with the parental and control cell lines. Experimental control cell lines, Hepswx and CHwt were transfected with the empty vectors, pcEF321swxneo, described elsewhere by Harada et al. [35] and pcCAGswxneo. This latter plasmid obtained by inserting the CAG promoter cassette in pEF321neo vector linearized by Asp718 digestion; is a derivative of pCAGGS [36], containing the unique Swa I cloning site.

The pCI-NF90 construct (courtesy of Prof. Kumar A, George Washington University) containing the NF90 sequence under the control of CMV promoter has been described [34]. Transfection of the established HepG2 and CHL cell lines with pCI-NF90 was performed by using Fugene 6 (Roche) according to manufacturer's instructions and with Fugene 6/plasmid DNA ratio of 3:1.

### Immunofluorescence and FACS analysis

Detection and quantitation of RANTES protein expression in parental and transfected cell lines was performed by intracellular immunofluorescence and FACS analysis. Briefly, sub-confluent cultures were detached with trypsin-EDTA solution and washed in PBS. After fixation for 20 minutes at room temperature in 4% paraformaldehyde and washing with permeabilization solution (Perm/Wash Buffer, Becton Dickinson), 10^6 ^cells were suspended in 0.5 μg of mouse anti-human RANTES phycoerythrin-conjugated monoclonal antibody in Perm/Wash Buffer and were incubated for 15 minutes at room temperature. After washing in Perm/Wash Buffer cells were analysed by FACScalibur flow cytometer using CellQuest software. A minimum of 10,000 events were analysed for each sample.

### RNA isolation and semiquantitative RT-PCR analysis

To detect RANTES mRNA, total RNA was extracted from cultured cell lines by RNeasy kit (Qiagen) according to manufacturer's instructions. 500 ng of total RNA was reverse transcribed using random hexanucleotides and 10 U of transcriptor™ reverse transcriptase (Roche) according to manufacturer's instructions with minor modifications and with the addition of recombinant RNAsin ribonuclease inhibitor. The PCR amplification of the reverse transcribed RNA was carried out with 1.5 unit of Taq DNA polimerase (ABI Prism) in 50 μl reaction mixture containing 200 μM of each dNTPs, 5 mM MgCl_2_, 20 μM of specific primers and 1× reaction buffer. Primers used for RANTES mRNA amplification in HepG2 cell lines were 5'-GCTGTCATCCTCATTGCTAC-3' (sense) and 5'-TCCATCCTAGCTCATCTCCA-3' (antisense), which yield a specific 260 (237) bp product; cycling conditions for HepG2 transfected cell lines were 94°C, 5 minutes, 56°C, 90 seconds and 72°C, 120 seconds, followed by 28 cycles at 94°C, 30 seconds, 56°C for 90 seconds and at 72°C for 120 seconds. Primers used to amplify RANTES from Chang Liver cell lines were: 5'-ACGCCTCGTGTCATCCTCATT-3' (sense) and 5'-ACTCTCCATCCTAGCTCATCTC-3' (antisense), which amplify a (226) bp product. PCR conditions were same as those described for HepG2 except MgCl_2 _concentration reduced to 1.5 mM and primers concentration to 0.1 μM. Cycling conditions were modified as follows: 94°C 4 minutes, 94°C 1 minute, 55°C 1 minute, 72°C 1 minute, for 40 cycles followed by 72°C 8 minutes. β-actin amplification in parallel samples was performed as controls by using following primers: 5'-ATCTGGCACCACACTTCTACA-3' (sense) and 5'-GTTTCGTGGATGCCAACAGGACT-3' (antisense) (550 bp product). Cycling conditions for β-actin were 94°C 4 minutes, 94°C 1 minute, 50°C 1 minute, 72°C 1 minute, followed by elongation at 72°C 8 minutes. In each experiment, possible DNA contamination was determined by a control reaction in which reverse transcriptase was omitted from the reaction mixture. For semi-quantitative RT-PCR analysis of RANTES, serial dilution of the cDNA samples was used. Amplified PCR fragments were analysed by electrophoresis in a 2% agarose gel.

### Real-Time quantitative PCR analysis

Real-Time quantitative PCR assessment of RANTES gene expression in Hep39 cell lines was performed using the ABI PRISM 7000 Sequence Detector system (Applied Biosystem). 800 nanograms (ng) of total RNA were *in vitro *reverse transcribed as described above for RT-PCR analysis. A portion of the resulting cDNA corresponding to 80 ng of *in vitro *transcribed RNA was subjected to Real-Time quantitative PCR that was performed in triplicate using TaqMan chemistry with primers and probe sets from the Assay-on-Demand list (Applied Biosystem). The standard curve of the target RANTES gene was compared to the standard curve of β-microglobulin gene used as endogenous control reference; calculation of the slope of the log (ng RNA) *vs *ΔCt was always <0.1. Fold reduction was then calculated by the ΔΔCt method after normalization of the mRNA level to the values of β-microglobulin.

### Transient transfection and luciferase reporter assay

2.5 μg of human RANTES reporter construct, pRANTES-Luc, (courtesy of Dr. Michael Gale), was transiently transfected by Fugene6 (Roche) in HepG2 and Chang Liver cell lines constitutively expressing HCV core protein, as well as in parental and the empty vector transfected control cells. To examine the effect of NF90 on RANTES activity 2.5 μg of the pCI-NF90 plasmid DNA was co-transfected with pRANTES-Luc into the liver cell lines. As transfection control, pSV β-galactosidase vector (1.0 μg) was co-transfected into the cells to monitor transfection efficiency. 48 h post-transfection, cell lysate was prepared and luciferase quantifications were performed using commercial reagents (Promega) and measuring in a Lumat LB 9501 Berthold Luminometer. Results are expressed as relative light units (RLU), calculated as the ratio of luciferase to β-galactosidase activities within each sample.

### SDS-PAGE and immunoblotting analysis of IRF-1 and IRF-7

Total proteins (50 μg) were diluted in 2× sample buffer, separated by 10% SDS-PAGE and transferred onto PVDF membranes. After incubation in blocking buffer containing 5% milk, membranes were incubated overnight at 4°C with specific polyclonal antibodies anti-IRF-1 or IRF-7 (Santa Cruz). The membranes were subsequently incubated with HRP-conjugated anti-rabbit IgGs and immunodetection was realized by ECL (Pierce) followed by autoradiography.

### Statistical analysis

The Student's t test and Fisher's exact test were used for comparison of the difference in RANTES expression between Hep39 and CH39 and their relative controls Hepswx and CHWT as well as to compare the luciferase activity of RANTES promoter in the two cell lines expressing HCV core protein. Differences were considered significant when *p *values were <0.05. Statistical analyses were performed using SPSS software (SPSS Inc).

## Results

### Effect of HCV core protein on RANTES protein

**Figure 1:**
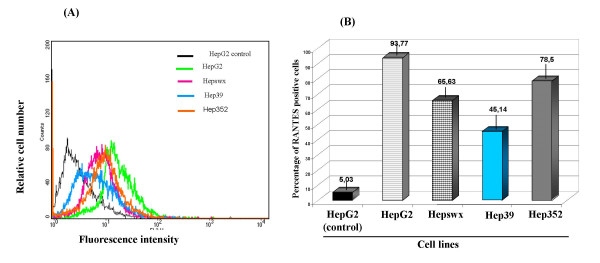
**Immunofluorescence and FACS analysis of RANTES expression**. (A) HepG2 cell lines were stained with anti-human RANTES mAb PE conjugated and processed for FACS analysis. (B) Percentage of cells positive for RANTES expression was plotted in columns. HepG2 control indicates parental cell line stained with an antibody isotypically related to anti-RANTES and used as negative control; HepG2 indicates parental cell line, Hepswx vector transfected, Hep39 core expressing cells, Hep352 cell line expressing C-E1-E2-NS2-NS3. One representative experiment out of three independently performed is shown; the standard deviation is indicated by the error bars in B plot; statistical significance (p < 0.05) was determined by Student's t-test for differences.

**Figure 2:**
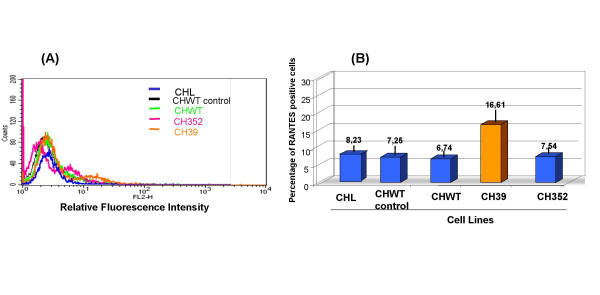
**Immunofluorescence and FACS analysis of RANTES expression**. (A) Chang Liver cell lines were stained with anti-human RANTES mAb PE conjugated and processed for FACS analysis. (B) Percentage of cells positive for RANTES expression was plotted in columns. CHWT control indicates vector transfected cells stained with an antibody isotipycally related to anti-RANTES and used as negative control cell line; CHWT indicates same cell line stained with anti-RANTES mAb; CHL are the parental Chang Liver cell line; CH39: core expressing cells; CH352: cells expressing C-E1-E2-NS2-NS3. One representative experiment out of three independently performed is shown; the standard deviation is indicated by the error bars in B plot; statistical significance (*p *< 0.05) was determined by Student's *t*-test for differences.

Considering the important role of chemokine signaling in regulating physiological events associated with chronic HCV infection, the effect of HCV core protein on RANTES expression was evaluated by quantitative immunofluorescence and flow cytometry in the two liver derived cell lines, HepG2 and Chang Liver, constitutively expressing HCV core protein. Results shown in Figures 1A and 1B indicated a twofold decrease in fractions of cells expressing RANTES protein for cells transfected with HCV core protein, as indicated by comparing percentage of positive HepG2 cell line controls (93.77% ± 0.8%) or the cells transfected with the empty vector (65.63% ± 1.1% in Hepswx) with the HCV core protein expressing cells (45% ± 2.6% in Hep39). Reduction in RANTES expression was detected also in Hepswx cells as compared to the parental cell line, suggesting a possible contribution of constitutive transfection on RANTES level. However the differences in percentage of cells positive for RANTES expression in Hep39 as compared to Hepswx cell lines was statistically significant (p < 005). However in Hep352 cell line (expressing core-E1-E2-NS3 proteins) percentage of RANTES positive cells (78,5 ± 1.5%) was close to that in control cell lines. In contrast, flow cytometry quantitation of RANTES protein in CH39 cell lines showed (Figures 2A and 2B) a statistically significant 2.5 fold increase in percentage of cells positive for RANTES expression, as compared with the CHWT control cells (16.6% ± 2.9% *vs*. 6.7% ± 1.2%, p < 0.05). In CH352 cell lines the fraction of RANTES positive cells (7.5% ± 0.7%) was similar to the controls. This latter result and that from Hep352 cells suggest a possible role of HCV structural E1 and E2 proteins or non-structural proteins NS2-NS3 in counteracting the effect of the core protein in RANTES induction. The results described above suggest that the effect of HCV core protein on the expression of RANTES may be regulated differently in liver cells derived from transformed hepatocytes and the non-transformed fibroblast cell lines.

### Analysis of HCV on RANTES mRNA

**Figure 3:**
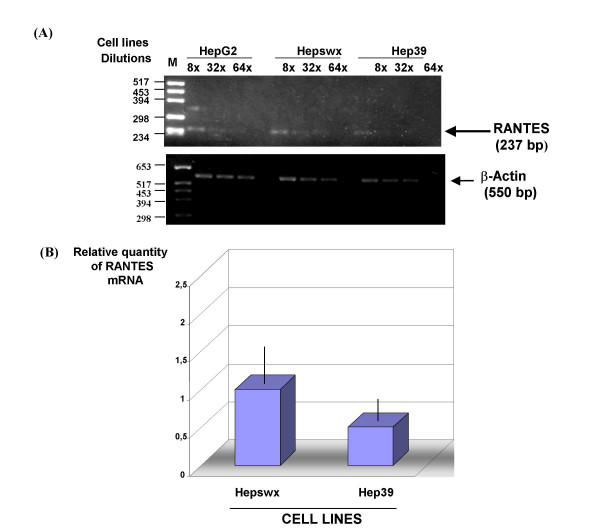
**Quantitative RT PCR analysis of RANTES mRNA**. (A) Serial dilution of cDNA samples (1:8, 1:32, 1:64 indicated as 8×, 32×, 64×) were prepared and subjected to PCR amplification. Lower panel shows β-actin mRNA amplification which was used as endogenous control. (B) Real-Time quantitative PCR analysis using RANTES primers. Data were normalized by the level of β2-microglobulin mRNA expression in each sample and are shown as the relative expression unit. Shown are the means ± standard deviations of two independent experiments with three replicates/sample in each experiment.

**Figure 4:**
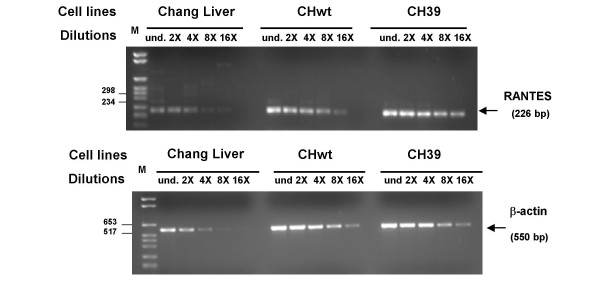
**Detection of RANTES transcripts by semi-quantitative RT-PCR**. cDNA samples from Chang Liver (CHL) cell lines transfected for constitutive expression of HCV proteins were serially diluted (1:8, 1:32, 1:64 indicated as 8×, 32×, 64×) and subjected to PCR analysis. In lower panel levels of endogenous β-actin mRNA are shown as control.

We analysed RANTES mRNA expression in the liver cell lines by RT-PCR using specific primers. Results showed (Figures 3 and 4) expected size band of RANTES cDNA, 257 and 268 bp respectively in HepG2 and in Chang Liver cell lines. Using similar quantity of cDNA (500 ng) for each sample from PCR amplification we made serial dilutions of RANTES cDNA to demonstrate that RANTES transcripts levels were decreased in Hep39 cell lines compared to the controls (Hepswx and HepG2) (Figure 3(A) upper panel). In order to corroborate these results, Real-Time quantitative PCR analysis was performed to determine relative RANTES mRNA levels in Hepswx and Hep39 cell lines. Results shown in Fig 3(B) indicated that in Hep39 cell lines RANTES mRNA level was 50% less than in Hepswx cell lines. Conversely, RANTES transcripts levels were increased by fourfold in CH39 cell lines, expressing HCV core protein (Figure 4), as compared to the control CHWT, and up to eightfold compared to parental CHL cells (Figure 4, upper panel). These results are consistent with the data presented above which examined the effect of core on RANTES protein expression, and suggest that HCV core protein affects RANTES chemokine expression in liver cell lines differentially.

### Effect of HCV core protein on RANTES promoter

**Figure 5:**
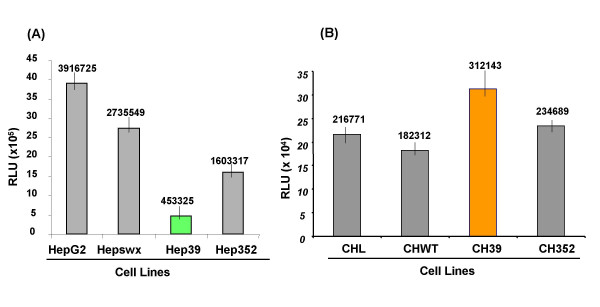
**RANTES promoter activity by luciferase assay**. Analysis of RANTES promoter activity in (A) HepG2 and (B) Chang Liver (CHL) cell lines constitutively transfected with empty vector (Hepswx and CHWT), or expressing HCV core protein (Hep39 and CH39) or C-E1-E2-NS2-NS3 (Hep352 and CH352). Results are expressed as the relative light units (RLU) of luciferase activity in each cell extract that was equalized for each sample. Data shown are the means of two independent transfections and three aliquots of cell extracts were analyzed in each experiment. The standard deviation is indicated by the error bars; statistical significance (*p *< 0.05) was determined by Student's *t*-test for differences.

We asked whether the modulation in RANTES expression by HCV core protein could be regulated at the transcriptional level. We carried out transient co-transfections with RANTES promoter-luciferase plasmid DNA in HepG2 and Chang Liver cell lines constitutively expressing core and core-E1-E2-NS2-NS3 proteins of HCV. Inhibition of RANTES promoter activity (Figure 5A) was observed in two different clones of Hep39 cells, with fourfold reduction (in RLU values) detected in Hep39 cells as compared to Hepswx cell lines. Interestingly, in Hep352 cell line a 3,5 fold induction of RANTES promoter was detected in comparison to Hep39 cell line that suggested a counteracting effect of the structural E1-E2 proteins or non-structural proteins NS2-NS3 toward the core protein inhibition on RANTES promoter also in HepG2. Conversely, in CH39 cells about twofold induction of RANTES promoter was detected (Figure 5B). The differences in RLU values among the cell lines in comparison to the controls were statistically significant according to the *p *value (<0.05) in a *t *Test's. Consistent to RANTES protein expression in CH352 the promoter of RANTES was not induced as in CH39, suggesting that the E1-E2 structural proteins or non-structural proteins NS2-NS3 may counteract the effect of the core protein on RANTES promoter activity.

These results are consistent with the effect of HCV core protein on RANTES mRNA levels and suggest that the core protein regulates RANTES expression by influencing its promoter activity.

### IRF-1 and IRF-7 expression in HepG2 and Chang liver cell lines

**Figure 6:**
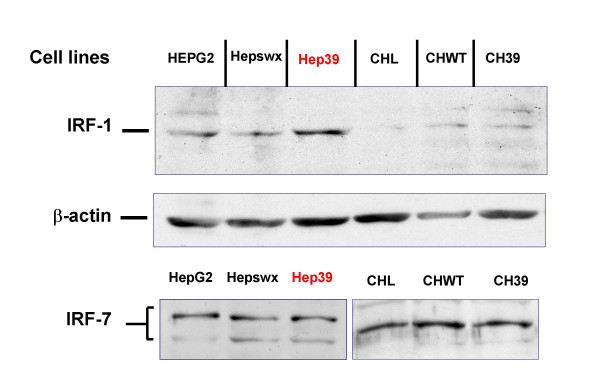
**Immunoblotting analysis of IRF-1 and IRF-7**. IRF-1 and IRF-7 were detected by Western blot analysis, as described in Material and Methods section, in controls HepG2 and Hepswx as well as in Hep39 cell lines and in Chang Liver, CHWT and CH39 cell lines; CTR+ is IRF-7 positive control cell lysate.

It has been reported that interferon response factors (IRF-1, -3, -7) influence RANTES promoter activity and RANTES expression in different cell lines [28,37]. We examined IRF-1 and -7 expression in the two liver cell lines (HepG2 and CHL) in order to correlate differential expression of the IRFs with the differences in RANTES induction between HepG2 and Chang Liver cells mediated by HCV core protein. The results of immunoblotting analysis (Figure 6) indicate that IRF-7 was equally expressed in either Hepswx and parental CHL cell lines (Figure 6, lower panel, lanes 1 and 3, respectively) and IRF-7 expression and phosphorylation were unaffected by the HCV core protein in the two cell lines under study, since no differences in the pattern of IRF-7 bands were detected in Hep39 or in CH39 cell transfectants (Figure 6 lower panel, lanes 2 and 4, respectively). With regard to IRF-1 expression, results of immunoblotting analysis indicated increased IRF-1 level in Hep39 cell lines (Figure 6, upper panel, lane 3); in contrast, IRF-1 was barely detectable in Chang Liver cell transfectants (Figure 6, lanes 5–7).

These results suggest that stable expression of HCV core protein induces IRF-1 expression in human hepatoblastoma cell line. Differential basal expression of IRF-1 between HepG2 and Chang Liver cell could be, at least in part, responsible for differential modulation of RANTES.

**Figure 7:**
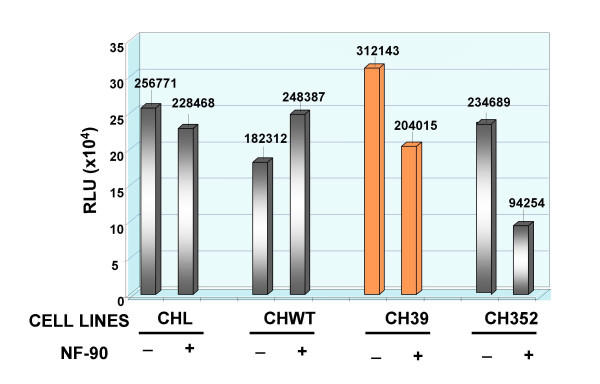
**Effect of NF90 on RANTES promoter activity in Chang Liver cell lines**. Effect of NF90 protein expression on RANTES promoter activity in controls CHL and CHWT cell lines and in CH39 and CH352 expressing HCV proteins was examined by co-transfection experiments as described in Material and Methods section. Results are expressed as the relative light units (RLU) of luciferase activity in each cell extract that was equalized for each sample. Data shown are the means of two independent transfections and three aliquots of cell extracts were analysed in each experiment. The standard deviation is indicated by the error bars; statistical significance (*p *< 0.05) was determined by Student's *t*-test for differences.

**Figure 8:**
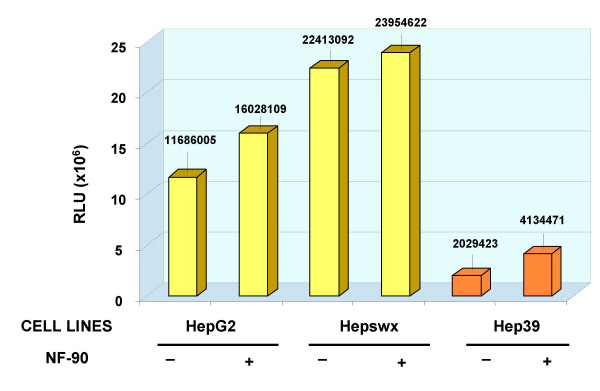
**Effect of NF90 on RANTES promoter activity in HepG2 cell lines**. Effect of NF90 protein expression on RANTES promoter activity in controls HepG2 and Hepswx cell lines as well as in Hep39 was investigated by co-transfection experiments as described in Material and Methods section. Results are expressed as the relative light units (RLU) of luciferase activity in each cell extract that was equalized for each sample. Data shown are the means of two independent transfections and three aliquots of cell extracts were analyzed in each experiment. the standard deviation is indicated by the error bars; statistical significance (*p *< 0.05) was determined by Student's *t*-test for differences.

Since NF90 is known to induce the transcriptional program of IFN response genes which is partly responsible for its interference with virus replication, we sought to examine whether the expression of NF90 affected the modulation of RANTES promoter in HCV core protein expressing cell lines as part of the anti-viral effect of NF90. The results in Chang Liver cells showed that NF90 countered the RANTES promoter activation mediated by the core protein, either in CH39 (1.5 times reduction as compared to NF90 controls, Figure 7), or in CH352 cells (the NF90-mediated inhibition was about 7 folds, Figure 7, right panel). In HepG2 cells or in Hepswx cell lines, the effect of NF90 on RANTES promoter was irrelevant (Figure 8). In Hep39 cells however, co-transfection with NF90 increased RANTES-promoter luciferase activity by twofold (Figure 8, right panel). The differences observed were statistically significant according to the *t *Test's (p < 0.05). The results suggest that NF90 expression could reverse the effect of HCV core protein on RANTES promoter either in HepG2 or in Chang Liver cell lines.

## Discussion

Results described in this study indicate that HCV core protein can either induce or inhibit RANTES chemokine expression in cell lines, depending on the cell type, arguing that RANTES promoter activity may be differentially regulated based on the availability of cell type specific transcription factors. Recruitment of virus-specific T cells to the liver is a critical step for HCV clearance. Chemokines such as RANTES, play a pivotal role in this process since they mediate lymphocytes migration. In addition, it has been reported [18], that RANTES/CCR5 expression is important in regulating relevant pathological events in the course of chronic hepatitis C infection; as well, intrahepatic expression of RANTES is positively related to the severity of hepatic inflammation in chronic hepatitis C. These results highlighted the significant role of this chemokine in HCV-induced liver disease. Consistent with recently reported observation [18], we found that HCV core protein variously modulated RANTES expression, by inducing its promoter. As discussed in the present study, opposite effects of HCV core protein on RANTES expression correlates with the different origin of the cell lines. HepG2 which is a human hepatoblastoma cell line with biochemical properties similar to hepatocytes, and Chang Liver cells are non-transformed human fibroblasts of liver origin. Immunohistochemical studies *in vivo *have indicated that RANTES and IP-10 chemokines are expressed in hepatocytes and that RANTES secretion increased in chronic hepatitis with a positive correlation to histological index of HCV related liver disease [16]. Similar to other cancers (ovary and colorectal tumors) chemokines, which contribute to lymphocyte recruitment at tumor sites and to immune-mediated attack [38] have been reported to be secreted by hepatocellular carcinoma (HCC) as well. Interestingly, it has been reported that a mammary murine carcinoma cell line expressing low levels of RANTES showed a decreased growth rate *in vivo *[8]. The reported evidences so far have pointed to a possible dual role of tumor-derived RANTES in tumor growth suggesting that inhibition of chemokine secretion by tumors could be a mechanism to evade immune attack. HepG2 cell line, a human hepatoblastoma cell line with hepatocytes characteristics, has been extensively investigated for the mechanisms of hepatocarcinogenesis; with regard to RANTES secretion HepG2 cell line behaves as tumor cells, whereas Chang Liver cells, a fibroblast-like cell line that is immortalized but without tumor phenotype, behave as non tumor cells with regard to the RANTES expression. The regulation of RANTES promoter by HCV proteins in these two liver cell types most likely reflects the available levels of the required transcription factors in these cell lines. The difference in the effects of HCV proteins on RANTES promoter is not due to their cellular localization, since the core protein is expressed in the cytoplasm of both HepG2 and the Chang Liver cells (data not shown). Since both the Hep39 and CH39 cell lines we utilized in the present study express full length (p21) HCV core protein, our data exclude the possibility that a truncated form of HCV core protein, which could migrate to the nucleus and affect NFk-B pathway (known to regulate expression of RANTES), could have influenced the results.

The effects of HCV envelope proteins on RANTES secretion are multifaceted. Recently, Soo et al. demonstrated that the intracellular co-expression of structural proteins E1-E2 suppress the HCV core-mediated activation of RANTES promoter reporter constructs in HeLa cells [18]. In contrast, extracellular binding of HCV E2 to CD81 has been shown to increase RANTES secretion [20]. We found, that co-expression of the core protein with E1-E2 and non-structural NS2-NS3 proteins of HCV counteracts the effect of HCV core expressed alone with respect to RANTES expression in both cellular systems. Thus, in line with Soo and colleagues, our results suggest that E1-E2 proteins counteract the effect of HCV core protein on RANTES expression, possibly by direct interaction of E1-E2 with the core protein in the cytoplasm. However, the role of the non-strucutral proteins NS2 and NS3 remains to be clarified in this context.

Expression of RANTES promoter has been reported to be regulated by IRF-3 and -7 cooperatively with NF-kB in U937 and in human kidney 293 cell lines as well as, in mouse macrophages, by IRF-1 which can bind to RANTES promoter [28,37]. The HCV core protein expression in Huh7 and HeLa cell lines is able to induce IRF-1 mRNA but it does not influence IRF-3 expression and phosphorylation [[29], personal unpublished observations]. Consistent with the data from Miller and co-workers [29] we found that HCV core protein induces expression of IRF-1 in human hepatoblastoma cell lines (HepG2), but not in human fibroblast of liver origin (CHL, where IRF-1 is slightly expressed in parental cells). We suggest that the available level and different basal expression of IRF-1 between HepG2 and CHL cell lines could explain, at least in part, the opposite effect of HCV core protein on RANTES induction in the two cell lines examined.

NF90ctv is a nuclear transcription factor belonging to double-stranded RNA (dsRNA)-binding proteins [33], which is involved in the life cycle of bovine diarrhea virus (BVDV) [33] and can activate interferon response genes and inhibit HIV-1 replication [34]. Interestingly, results obtained from present study indicated that NF90 protein has the ability to counter the effect of HCV core protein mediated RANTES expression in both cell types examined, suggesting its ability to counter virus mediated effect on host cell antiviral response. The mechanisms underlying its role are under investigation.

## Conclusion

HCV core protein have opposite effects on the expression of RANTES in different cell types *in vitro*, possibly reflecting a similar scenario in different microenvironments *in vivo*.

Based on the data presented here we speculate that cell type specific factors play a fundamental role in RANTES expression. It is conceivable that core protein, which is expressed early in HCV infection *in vivo *and whose expression is maintained during chronic phase and in HCC cells, may have the opposite effects, influencing T cell trafficking in the liver through either the inhibition/induction of RANTES. Our data suggest a role, that needs to be confirmed *in vivo *for viral (HCV) proteins in contributing to immune evasion by tumor cells.

## Competing interests

The author(s) declare that they have no competing interests.

## Authors' contributions

AR: guided study concept and design, data analysis and interpretation, writing of manuscript.

MF and IG: data acquisition and data analysis.

AK: critical revision of the manuscript, provision of NF90 constructs.

MR: guided study concept and reviewed final submission.

All authors read and approved the final manuscript.

## Pre-publication history

The pre-publication history for this paper can be accessed here:


